# Efficacy of osteoporosis pharmacological treatments in men: a systematic review and meta-analysis

**DOI:** 10.1007/s40520-023-02478-9

**Published:** 2023-07-03

**Authors:** Charlotte Beaudart, Céline Demonceau, Shaun Sabico, Nicola Veronese, Cyrus Cooper, Nicholas Harvey, Nicholas Fuggle, Olivier Bruyère, René Rizzoli, Jean-Yves Reginster

**Affiliations:** 1 WHO Collaborating Center for Public Health aspects of musculo-skeletal health and ageing, WHO Collaborating Center for Epidemiology of Musculoskeletal Health and Aging, Division of Public Health, Epidemiology and Health Economics, University of Liège, Belgium, Avenue Hippocrate 13, CHU Bât B23, 4000 Liège, Belgium; 2 Biochemistry Department, College of Science, Chair for Biomarkers of Chronic Diseases, King Saud University, Riyadh, 11451 Saudi Arabia; 3 Geriatric Unit, Department of Internal Medicine, Geriatrics Section, University of Palermo, via del Vespro, 141, 90127 Palermo, Italy; 4 MRC Lifecourse Epidemiology Centre, University of Southampton, Southampton, SO16 6YD UK; 5 Service of Bone Diseases, Geneva University Hospitals and Faculty of Medicine, Hopitaux Universitaires Geneve, Geneva, Switzerland

**Keywords:** Osteoporosis, Men, Bone mineral density, Fractures

## Abstract

**Introduction:**

The objective of this systematic review and meta-analysis is to systematically identify and review the efficacy of pharmacological treatments in men with osteoporosis.

**Methods:**

Medline (via Ovid) and Cochrane CENTRAL were searched up to May 2023 for any randomized controlled trial (RCT) evaluating the efficacy of osteoporotic treatment on the evolution of Bone Mineral Density (BMD) and incidence of fractures of men suffering from primary osteoporosis. If at least two studies used the same pharmacological treatment and evaluated the same outcome, a random effect model meta-analysis was applied to reported pooled mean difference (MD) and 95% confidence interval (CI).

**Results:**

From the 1,061 studies identified through bibliographic search, 21 RCTs fitted the inclusion criteria. Bisphosphonates (k = 10, n = 2992 men with osteoporosis) improved all three BMD sites compared to placebo; lumbar spine: MD + 4.75% (95% CI 3.45, 6.05); total hip: MD + 2.72% (95% CI 2.06; 3.37); femoral neck: MD + 2.26% (95% CI 1.67; 2.85). Denososumab (k = 2, n = 242), Teriparatide (k = 2, n = 309) and Abaloparatide (k = 2, n = 248) also produced significant improvement of all sites BMD compared to placebo. Romosozumab was only identified in one study and was therefore not meta-analysed. In this study, Romosozumab increased significantly BMD compared to placebo. Incident fractures were reported in 16 RCTs but only four reported fractures as the primary outcome. Treatments were associated with a lower incidence of fractures.

**Conclusions:**

Medications used in the management of osteoporosis in women appear to provide similar benefits in men with osteoporosis. Therefore, the algorithm for the management of osteoporosis in men could be similar to the one previously recommended for the management of osteoporosis in women.

**Supplementary Information:**

The online version contains supplementary material available at 10.1007/s40520-023-02478-9.

## Introduction

Osteoporosis is a systemic skeletal disease characterized by low bone mass and microarchitectural deterioration of bone tissue leading to increased bone fragility and fracture susceptibility [1]. Bone fractures are a major health consequence of osteoporosis leading to an increased risk of mortality, disability, loss of independence and increased medical costs [2–4]. Worldwide, 23% of women and 12% of men have osteoporosis, with the prevalence increasing significantly with age [5]. At the age of 50, the lifetime risk of experiencing a fracture is about 20% for a man whilst it is close to 50% for a woman [6]. As compared with a woman, fragility fractures in men are associated with more morbidities, greater need for long-term care, more disabilities and higher mortality [7, 8]. Thus, osteoporosis in men represents a significant public health threat [9]. For example, in the European Union, the total health burden due to fractures in men in 2010 was estimated to be 384,000 lost quality-adjusted life years (QALYs) and projected to increase to 491,000 QALYs lost in 2025 [10].

Numerous pharmacological therapies have been proposed to reduce fracture risk in patients with osteoporosis. In a recent network meta-analysis including 108,797 individuals from 79 randomized controlled trials, Shen et al. reported that pharmacological therapies like alendronate zolendronate, risedronate, ibandronate, denosumab, abaloparatide, teriparatide and romosozumab were all effective treatments to reduce the risk of fractures [11]. The majority (i.e. 86%) of the trials included in this meta-synthesis sampled postmenopausal women. As a considerable amount of evidence is convergent regarding the efficacy of the above-mentioned therapies in women [11–15], it is now accepted from regulatory agencies to grant marketing authorization of these drugs for men with osteoporosis following the conduct of bridging studies [16, 17]. In these studies, the primary outcome is no longer the risk of fracture but rather an increase of bone mineral density (BMD) similar to that observed in women [18, 19]. Requirements for this bridging concept include the use of the same formulation, dose and route of administration; the inclusion of a male population with a fracture risk of a similar magnitude compared with that of the postmenopausal women studied; and demonstration of similar changes in BMD in a 1‐year study [16, 17].

Although the efficacy of pharmacological treatment has been less studied in men compared to women, some meta-research studies have been published in the last few years to summarize the available evidence in men [20]. In 2015, Chen et al. [21] published a network meta-analysis aiming to provide a hierarchy of eight different drugs for their impact on bone mineral density of men and included 13 randomized controlled trials published until 2014. The authors reported that zoledronate had the most significant effect on increasing lumbar spine bone mineral density followed by alendronate, the combination of teriparatide + risedronate, risedronate alone, teriparatide alone, strontium ranelate, ibandronate and parathyroid hormone. Later in 2017, Nayak et al. [22] included 22 studies published up to 2016 in a meta-analysis aimed at assessing the evidence for the efficacy of treatment to reduce osteoporotic fracture risk in men. The authors concluded that bisphosphonates are effective in reducing the risk of vertebral fractures in men with osteoporosis but acknowledged that studies in this area are still required to provide robust evidence.

As some new trials have been published in the last 7 years, there is a need to update these works in order to provide the most up-to-date evidence-based data on the efficacy of osteoporosis pharmacological treatments in men. Therefore, the objective of this systematic review and meta-analysis is to systematically identify and review the efficacy of osteoporosis interventions in men.

## Methods

The proposed systematic review was conducted and reported in accordance with the Preferred Reporting Items for Systematic Review and Meta-analysis (PRISMA) 2020 [23]. A protocol has been developed and published in both PROSPERO (ID 395481) and Open Science Framework (https://osf.io/wqy3n/).

The research question can be summarized using the PICOs format: P (Population): Male adults (> 18 years) with primary osteoporosis (i.e. age-related osteoporosis); I (Intervention) Any osteoporosis treatment; C: Placebo or other active drugs; Outcome: evolution of bone mineral density (BMD) in three sites; lumbar spine (LS), total hip (TH) and femoral neck (FN); incidence of vertebral (V) and non-vertebral (NV) fractures.

### Literature search

Medline (via Ovid) and Cochrane CENTRAL databases were searched in May 2023 for any randomized controlled trial evaluating the efficacy of osteoporotic treatment on the evolution of BMD and incidence of fractures of men suffering from primary osteoporosis. For convenience of translation, the search was limited to English and French studies [24]. A combination of terms of Medical Subject Headings (MeSH) and keywords was used in the search strategy (the complete search strategies for both databases are available in Appendix A1).

Additionally, a manual search within the bibliography of relevant papers was performed in order to complete the bibliographic search. Forward references searching of included studies was also conducted using Web of Science to identify other research that has referenced any article of interest. Previous systematic reviews and meta-analyses on the same topic were also searched for backward/forward referencing. Clinical trial registries (www.clinicaltrial.gov) were also searched for potential unpublished studies and experts in the field were contacted to obtain their opinions about the search strategy and the included papers. Those experts were asked to provide any missing studies or grey literature they were aware of. Finally, industry members developing osteoporosis treatments were contacted in order to obtain unpublished data on their products.

The search results from the electronic sources and hand searching were imported into Covidence software for data management.

### Study selection

All identified articles were screened for their eligibility by two independent reviewers (C.B., C.D. or S.S) first based on their titles and abstracts and second, based on their full texts. Inclusion criteria (Table 1) guided the study selection process. During both stages, disagreements were resolved by consensus.

**Table 1:** Inclusion criteria

PICO(S) criteria	
Patients	Male adults with primary (i.e. age-related) osteoporosis, with or without history of fracture
Intervention	Any osteoporosis treatment (Denosumab, alendronate, risedronate, ibandronate, abaloparatide, teriparatide, romosozumab, zoledronate) Both arms could include Ca and or vitamin D
Comparator	Placebo or any other active drugs (listed in interventions)
Outcome	Evolution of BMD (Lumbar spine (SP), Total Hip (TH), femoral neck (FN)) Incidence of vertebral fractures (VF) and non-vertebral (NVF) fractures
Study design	Randomized controlled trials

Studies with secondary causes of osteoporosis (cancer-related, hypogonadism and corticosteroid-induced osteoporosis), studies published in other languages than French and English [24], not original studies (case reports, review, letters to the editors, conference abstracts, opinion pieces) and protocols were excluded.

### Data extraction

Data were extracted by one independent reviewer according to a standardized data extraction form pretested on a sample of 4 studies. A second reviewer checked data extraction for accuracy. The following data were extracted: information related to the reference (author, year of publication, journal, funding, conflicts of interest), information related to the study design (design of intervention, groups, sample size, analysis per protocol or intention-to-treat), information related to the treatment (type of treatment, dose, route of administration, length of follow-up) and information related to the outcome (mean BMD, incidence of fracture in each groups). Authors of individual papers were contacted in case of any missing information.

### Quality appraisal

The Revised Cochrane risk-of-bias tool for randomized trials (RoB2) was used to appraise the quality of individual studies [25]. This tool assesses 5 domains: randomisation process, deviation from intended interventions, missing outcome data, measurement of the outcome and selection of the reported results. A judgment per domain and an overall judgment or risk of bias was provided and studies were rated as having low risk of bias, some concerns or high risk of bias. When a study did not publish a protocol, domain 5 was automatically rated as “some concerns”. When a study was not double-blinded, domain 2 was automatically rated as “some concerns”.

Each study was evaluated independently by two reviewers. Disagreements were resolved by consensus or with the help of a third expert reviewer.

### Grading the evidence

For all significant associations determined by meta-analyses, the evidence derived from RCTs was evaluated using the GRADE (Grading of Recommendations, Assessments, Development and Evaluation) assessment [26]. The evidence score started at high-quality evidence and was downgraded by one (i.e. moderate quality evidence), two (i.e. low quality evidence) or three levels (i.e. very low quality evidence) if one of the following pre-specified criteria was present: 1) Risk of bias (i.e. high risk of bias in more than 75% of the included studies; 2) Inconsistency (i.e. unexplained substantial heterogeneity I^2^ > 50%); 3) Indirectness (i.e. presence of factors that limit the generalizability of the results); 4) Imprecision (i.e. large 95%CI, recommendation altered if 95%CI represents the true effect), 5) Publication bias (i.e. small study effect p > 0.05 and significant impact on the estimator). Each meta-analysis outcome assessed was determined to be of very low, low, moderate or high certainty.

### Data synthesis

Results on the effect of treatments on (1) BMD sites and (2) incidence of fractures were presented. Unfortunately, because fractures were rarely reported as primary endpoint in the included studies and mostly presented as safety results, it was not considered appropriate to perform a meta-analysis on this outcome. Results regarding the effect of fractures were therefore only presented in a narrative form.

For the effect on BMD, if at least two studies using the same pharmacological treatment and evaluating the same outcome were available to be pooled in a meta-analytical model, a random effect model was applied to calculate a pooled Mean Difference for each BMD site. Separate meta-analyses were performed for each treatment. Additionally, anti-resorptive and anabolic agents were grouped together and the pooled MD for both types of treatment were obtained by random effect model. When possible, effect sizes from intent-to-treat analysis were used in our analyses. For studies reporting the outcomes for multiple follow-up time periods, the outcomes reported for the longest follow-up time period under treatment was used. Results were examined for heterogeneity using Cochran’s Q statistic and the I^2^ statistic. The Egger's regression asymmetry test was used to detect publication bias for meta-analysis including a sufficient number of studies. One-way sensitivity analyses were also conducted to evaluate the stability of the results when one study is removed at a time.

When data were not available in the right format or incomplete, authors of individual studies were contacted to obtain missing values. Authors were contacted twice with a one-month interval between contacts. If the missing data could not be obtained from the authors, different strategies to obtain the missing information were used: (1) application of the methods described in Sect. 7.7.3 of the Cochrane Handbook for Systematic Review [27] to obtain missing SD’s from SE, from p-values or 95% confidence intervals; (2) when no information was provided to obtain the missing SD’s, SD’s were extracted from another study with a similar sample size, (3) when only median and interquartile ranges were available, the formula proposed by Hozo et al. [28] to convert them into mean and SDs was used.

For all results, a two-sided p value of 0.05 or less was considered as significant. All analyses were performed using R Software and appropriate packages.

If a meta-analysis could not be performed, a narrative description of the results was provided.

## Results

### Studies characteristics and risk of bias evaluation

**Fig. 1:**
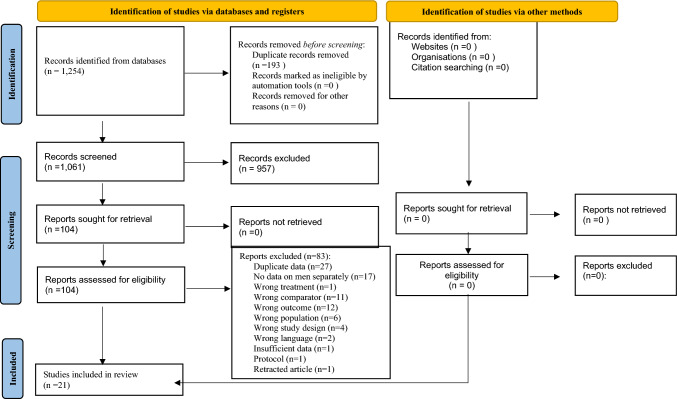
Preferred reporting items for systematic reviews and meta-analyses (PRISMA 2020) flowchart of study selection

A total of 1,254 references were identified through the search strategies applied on bibliographic databases in May 2023 2022. After removing duplicates, 1,061 references were assessed for eligibility based on their title/abstract. Among those references, 104 were assessed based on their full text and 21 RCTs met the inclusion criteria and were further included in this systematic review and meta-analysis [29–50] (Fig. 1). The list of excluded studies in the stage of full-text review as well as of the reason for exclusion is available on our Open Science Framework deposit (https://osf.io/wqy3n/).

Studies were published between 2003 and 2022. The number of male patients included in the studies varied from 20 in the study of Matsumoto et al. [51] to 1199 in the study of Boonen et al. [44]. Sixteen out of the 21 included RCTs (i.e. 76.2%) were double-blinded. The efficacy of 8 different treatments was investigated through those 21 RCTs: alendronate (n = 8), risedronate (n = 3), zoledronic acid (n = 3), ibandronate (n = 1), denosumab (n = 2), teriparatide (n = 5), abaloparatide (n = 2), romosozumab (n = 1). Most of the studies (n = 17, 81%) used placebo as comparator; other studies were head-to-head RCTs [35, 36, 41, 48] comparing the efficacy of two active drugs. The median length of treatment was 78 weeks (range 24 weeks [47] to 156 weeks [49]). In regards of outcomes, all studies reported measures of BMD (n = 21, 100%), with lumbar spine BMD defined as primary outcome in 14 studies (66.7%). Incidence of fracture was reported in 16 studies (76.2%) and only four studies defined the incidence of fracture as the primary outcome. In the other studies, incidence of fracture was reported either as a secondary outcome or as a safety measurement. Table 2 contains the detailed characteristics of the 21 RCTs included.

**Table 2:** Studies included in the SR/MA

Author, year	Study design	Country	Treatment	Comparator	No patient/Group	Length of interv	Outcomes	Funding
Boonen, 2009 [43]	RCT, double-blind, placebo controlled	Multicenter study^*a^	Risedronate 35 mg, daily oral administration + Ca (1 g) and vit D (400–500 IU), twice daily	Placebo + Ca (1 g) and vit D (400–500 IU), twice daily	G1: 191 G2: 93	104 weeks (2 years)	BMD: LS‡, Proximal Femur, Trochanter Fractures: VF, all fractures	Alliance for Better Bone Health, a partnership between Pr`octer & Gamble Pharmaceuticals and Sanofi-Aventis Pharmaceuticals
Boonen, 2011 [30]	RCT, double blind, placebo controlled	International	Zoledronic Acid 5 mg, yearly intravenous injection + Ca (1–1.5 g) and Vit D (400–800 IU), daily administration	Placebo + Ca (1–1.5 g) and Vit D (400–800 IU), daily administration	G1:248 G2: 260	104 weeks (2 years)	BMD: LS, TH Fractures: VF‡, NVF	Novartis Pharma
Boonen, 2012 [44]	RCT, double blind, placebo controlled	Europe, South America, Africa, and Australia	Zoledronic acid 5 mg, yearly intravenous injection + Ca (1 g) and Vit D (800–1000 IU), daily oral administration	Placebo + Ca (1 g) and Vit D (800–1000 IU)	G1: 588 G2: 611	104 weeks (2 years)	BMD: FN, TH Fracture, VF, Total fractures	Novartis Pharma
Czerwinski, 2022 [42]	RCT, double-blind, phase 3, placebo controlled	NR	Abaloparatide 80 µg, daily subcutaneous injection	Placebo	G1: 149 G2: 79	52 weeks (1 year)	BMD: LS‡, TH, FN Fractures	Radius Health, Inc (Radius)
Finkelstein 2003 [48]	RCT, Open-label trial, active controlled	Boston, USA	Alendronate 10 mg, oral daily administration (for 30 months) + Ca (1000–1200 mg) either by diet or supplementation and Vit D (400U), daily oral administration	1/ Human PTH (1–34) 40ug, daily subcutaneous injection (for 24 months) + Ca (1000–1200 mg) either by diet or supplementation and Vit D (400U), daily oral administration 2/ Combination of both	G1: 28 G2: 27 G3: 28	120 weeks	BMD: LS‡, TH, FN	Supported by grants from the National Institutes of Health
Gonnelli 2003[49]	RCT, Open-label trial	Italy	Alendronate 10 mg, oral daily administration + Ca (1000 mg) daily oral administration	No placebo. The control group received only Ca (1000 mg) daily oral administration	G1: 39 G2: 38	156 weeks (3-years)	BMD: LS, TH, FN	NR
Hwang, 2010 [47]	RCT, Open-label trial	Taiwan	Alendronate 70 mg, oral weekly administration + Ca and Vit D supplement, daily oral administration	No placebo. The control group only received Ca and Vit D supplement, daily oral administration	G1: 23 G2 23	24 weeks	BMD: LS‡, TH, FN	Merck Sharp & Dohme (MSD) and TTY Biopharm
Kurland 2000 [45]	RCT, double blind, placebo controlled	Columbia	Parathyroid hormone, 34-amino acid fragment of human PTH-(1–34) 400 IU + Ca (1500 mg) and Vit D supplement (400 IU), daily oral administration	Placebo (consisting of mannitol and citric acid) + Ca (1500 mg) and Vit D supplement (400 IU), daily oral administration	G1: 13 G2: 10	76 weeks(18 months)	BMD: LS, TH, FN Fractures: VF	Supported in part by FDA Grant FD-R001024, NIH Grants AR-39191 and M01-RR-00645, and Biomeasure, Inc. (Milford, MA)
Lewiecki 2018 [29]	RCT, double-blind, phase 3, placebo controlled	Thirty-one centres in Europe, Latin America, Japan, and North America	Romosozumab 210 mg, monthly subcutaneous injection + Ca (500–1000 mg) and vit D (600–800 IU), daily oral administration	Placebo + Ca (500–1000 mg) and vit D (600-800 IU), daily oral administration	G1: 163 G2: 82	52 weeks (1 year)	BMD: LS‡, TH, FN Fractures	UCB Pharma
Matsumoto 2022 [51]	RCT, double-blind, phase 3, placebo controlled	21 sites in Japan	Abaloparatide 80 µg, daily subcutaneous self-injections + Ca and Vit D supplement, daily oral administration	Placebo + Ca and Vit D supplement, daily oral administration	G1: 14 G2: 6	78 weeks	BMD: LS‡, TH, FN Fractures	Teijin Pharma Limited
Miller 2004 [32]	RCT, double blind	USA, Multicentre	Alendronate 70 mg, weekly oral administration + Ca as carbonate (500 mg) and Vit D (200 IU), daily oral administration	Placebo + Ca as carbonate (500 mg) and Vit D (200 IU), daily oral administration	G1: 109 G2: 58	52 weeks (1 year)	BMD: LS‡, FN Fractures	Merck
Nakamura 2014 [31]	RCT, double-blind, phase 3, placebo controlled	Japan	Denosumab 60 mg, subcutaneous injection every 6 months + Ca (600 mg) and vit D (400 IU), daily oral administration	Placebo + Ca (600 mg) and vit D (400 IU), daily oral administration	Males participants G1: 23 G2: 24	104 weeks (2 years)	BMD: LS, TH, FN Fractures‡	Daiichi-Sankyo Co Ltd, Tokyo, Japan
Orwoll 2000 [34]	RCT, double-blind, placebo controlled	20 centers in the United States and 10 other countries	Alendronate 10 mg, daily oral administration + + Ca (500 mg) and Vit D (400 IU), daily oral administration	Placebo + Ca (500 mg) and Vit D (400 IU), daily oral administration	G1:146 G2:95	104 weeks (2 years)	BMD: LS‡, TH, FN Fractures	Merck
Orwoll 2003 [37]	RCT, double-blind, placebo controlled	37 centers in 11 countries	Teriparatide 20ug, subcutaneous daily injection + Ca (1000 mg) and Vit D (400–1200 IU), daily oral administration	1/ Teriparatide 40ug, subcutaneous daily injection 2/ Placebo + Ca (1000 mg) and Vit D (400–1200 IU), daily oral administration	G1: 151 G2: 139 G3: 147	52 weeks (1 year)	BMD: LS‡, TH, FN	Eli-Lilly
Orwoll 2010 [35]	RCT, double-blind, active controlled	North America, Australia	Zoledronic acid 5 mg, yearly intravenous injection + Ca (1 g) and Vit D (800–1000 IU), daily oral administration	Alendronate 70 mg, oral daily administration + yearly intravenous injection of placebo + Ca (1 g) and Vit D (800-1000 IU), daily oral administration	G1: 154 G2: 148	104 weeks (2 years)	BMD: LS‡, TH, FN Fractures: VF	Novartis
Orwoll 2010 [33]	RCT, double blind, placebo controlled	USA	Ibandronate 150 mg, oral monthly administration + Ca (1 g) and vit D (400 IU) twice daily	Placebo + Ca (1 g) and vit D (400 IU) twice daily	G1: 85 G2: 47	52 weeks (1 year)	BMD: LS‡, TH, proximal femur Fractures: VF, Clinical fractures	Roche and GlaxoSmithKline
Orwoll, 2012 [38]	RCT, double-blind, phase 3, placebo controlled	Multicentre study (North America and Europe)	Denosumab 60 mg, sub cutaneous injection every 6 months (q6m) + Ca (≥ 1 g) and Vit D (≥ 800 IU), daily oral administration	Placebo + Ca (≥ 1 g) and Vit D (≥ 800 IU), daily oral administration	G1: 121 G2: 121	52 weeks (1 year)	BMD: LS‡, TH, FN	Amgen Inc
Qi 2021 [36]	RCT, Open-label trial, active controlled	China	Teriparatide 20 µg/day, daily subcutaneous injection + Ca and Vit D (dose not provided), daily oral administration	Alendronate 10 mg/day, oral daily administration + Ca and Vit D (dose not provided), daily oral administration	Males participants: G1: 50 G2: 50	52 weeks (1 year)	BMD: LS Fractures	NR
Ringe 2009 [39]	Prospective, match-control trial, Open-label trial	Germany	Risedronate 5 mg, oral daily administration + Ca (1,000 mg) daily and Vit D (800 IU), daily oral administration	No placebo group. If prevalent VF: alfacacidol (1 µg) + Ca (500 mg), daily oral administration If no previous VF: Vit D (1,000 IU) plus Ca (800 mg), daily oral administration	G1: 158 G2: 158	104 weeks (2 years)	BMD: LS, TH, FN Fractures: VF‡, NVF	None
Shimon 2005 [40]	RCT, double blind, placebo controlled	Israel	Alendronate 10 mg, oral daily administration + Ca (800 mg) and multivitamin containing 600 IU of Vit D, daily oral administration	Placebo + Ca (800 mg) and multivitamin containing 600 IU of Vit D, daily oral administration	G1: 11 G2: 13	52 weeks (1 year)	BMD: LS‡, FN Fractures	Merck
Walker 2013 [41]	RCT, double-blind, active controlled	Columbia	Risedronate oral 35 mg, weekly oral administration + daily subcutaneous injection of placebo + Ca (500 mg) and vit D (400 IU), daily oral administration	1/Teriparatide daily subcutaneous injection 20 µg + weekly oral administration of placebo + Ca (500 mg) and vit D (400 IU), daily oral administration 2/ Combination of both	G1: 10 G2: 9 G3: 10	78 weeks (18 months)	BMD: LS‡, TH, FN Fractures	Alliance for Better Bone Health, a partnership between Procter & Gamble Pharmaceuticals and Sanofi-Aventis Pharmaceuticals

‡ mentioned as primary outcome in analyses

a recruitment centres: Eastern and Western Europe, Lebanon, Australia, and the United States

*LS* lumbar spine, *TH* total hip, *FN* femoral neck, *BMD* bone mineral density, *VF* vertebral fractures, *NVF* non vertebral fractures, *Ca* Calcium, *Vit D*: vitamin D

Only one study presented a high risk of bias [36]. However, Boonen et al. 2012 [44], Orwoll et al. 2012 [38], Matsumoto et al. [51] were the only three studies rated as low risk of bias studies. All the other studies presented some concerns in regard to the risk of bias and mainly for Domain 5 (i.e. selection of the reported results). Indeed, most of the studies (i.e. 14/21, 66.7%) did not published an a priori protocol (Appendix A2).

Due to the number of studies available, five different meta-analyses were possible: (1) effects of any bisphosphonate versus placebo on BMD; (2) effects of alendronate versus placebo on BMD; (3) effects of risedronate versus placebo on BMD; (4) effects of teriparatide versus placebo on BMD and (5) effects of abaloparatide versus placebo on BMD. All the other associations with BMD as well as all associations between treatments and incidence of fractures were described narratively.

### Effect of bisphosphonates versus placebo on BMD

#### Any bisphosphonates (Alendronate, Risedronate, Ibandronate, Zoledronic Acid)

Ten studies, including 2992 men with osteoporosis, compared treatment with bisphosphonates to placebo [30, 32–34, 39, 40, 43, 44, 47, 49]. Meta-analytic synthesis was feasible for all investigated outcomes (LS BMD n = 9, TH BMD n = 9, FN BMD n = 8). Duration of treatment ranged from 6 months [47] in the study of Hwang et al. to three years in the study of Gonneli et al. [49].

**Fig. 2:**
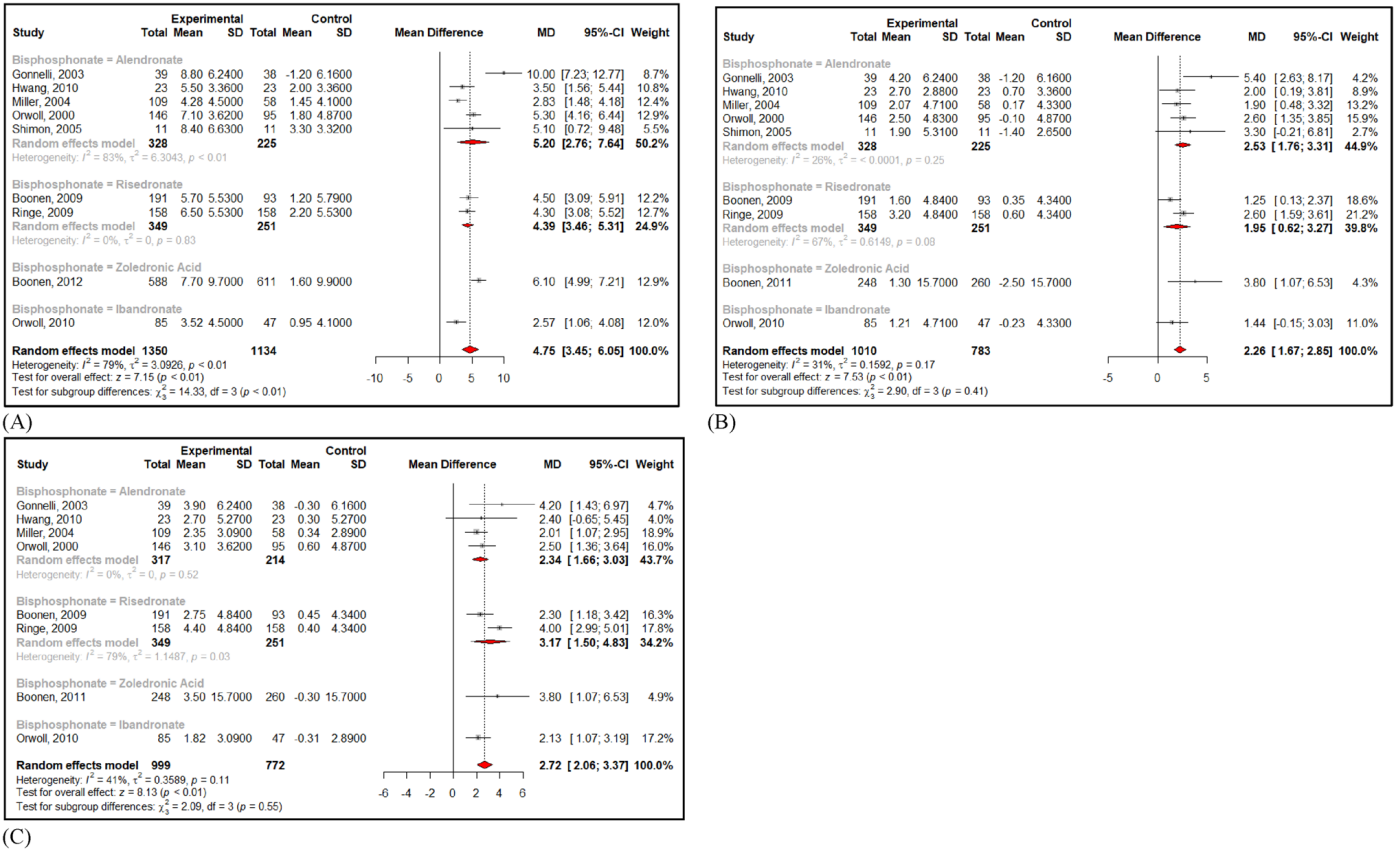
Effect of bisphosphonates on LS BMD (**A**) FN BMD (**B**) and TH BMD (**C**)

Compared to placebo, bisphosphonates significantly increased BMD at 3 sites (Fig. 2): LS BMD (Mean Difference of 4.75% (95% CI 3.45, 6.05), I^2^ = 79%), TH BMD (Mean Difference of 2.72% (95% CI 2.06; 3.37), I^2^ = 41%) and FN BMD (Mean Difference of 2.26% (95% CI 1.67; 2.85), I^2^ = 31%). No significant publication bias was found in those three meta-analyses (Egger test p > 0.05).

The one-leave-out analysis (Appendix A3) did not revealed the specific impact of any of the individual study on the pooled effect size.

#### Alendronate

Five studies, including 553 men with osteoporosis, compared alendronate to placebo [32, 34, 40, 47, 49]. Three studies provided daily 10 mg alendronate administration during, respectively 1 year [40], 2 years [34] and 3 years [49]. Two others provided weekly 70 mg alendronate administration during either 6 months [47] or 1 year [32]. Meta-analysis was possible for all investigated outcomes (LS BMD n = 5, TH BMD n = 4, FN BMD n = 5, VF n = 3, NVF n = 3). Compared to placebo, alendronate seems to significantly improve LS BMD with a MD of 5.2% (95% CI 2.76;7.64) (I^2^ = 83%, p < 0.01), TH BMD with a MD of 2.34% (95% CI 1.66–3.03) (I^2^ = 0%, p = 0.81) as well as FN BMD with a MD of 2.53% (95% CI 1.76;3.31) (I^2^ 26%, p = 0.25).

#### Risedronate

Two studies, including 600 men with osteoporosis treated during 2 years, compared risedronate to placebo [39, 43]. Boonen et al. provided once-weekly administration of 35 mg of risedronate or placebo whereas Ringe et al. provided daily administration of 5 mg of risedronate or placebo. Risedronate seems to be beneficial for all the investigate outcomes except the incidence of vertebral fractures (p = 0.25). A MD of 4.39% (95% CI 3.46;5.31) (I^2^ = 0%, p = 0.83) was found for LS BMD, a MD of 2.46% (95% CI 1.71;3.22) (I^2^ = 0%, p = 0.7) was found for TH BMD and a MD of 1.95% (95% CI 0.62;3.27) (I^2^ = 67%, p = 0.08) was found for FN BMD.

#### Zoledronic acid

Two studies, including 1,707 men with osteoporosis treated during 2-years, compared zoledronic acid to placebo [30, 44]. Because papers did not report similar outcomes, meta-analytic synthesis was not possible for zoledronic acid. In 2012, Boonen et al. 2012 [44] reported a significant improvement of LS BMD in 1,199 patients treated with yearly intravenous injection of 5 mg Zoledronic Acid (MD of 6.10%, 95% CI 4.99–7.21). A significant improvement of TH BMD (difference between groups of 3.8%, 95%CI 2.2–5.4) and FN BMD (difference between group of 3.1%, 95% CI 2.2; 5.4) was also demonstrated in a sample of 508 men with a recent hip fracture (Boonen et al. 2011 [30]).

#### Ibandronate

One study, including 132 men with osteoporosis treated for 1 year, compared ibandronate (150 mg, monthly) to placebo [33]. In this study, Orwoll et al. [33] reported a significant improvement of LS BMD (difference between groups of 2.58%, 95% CI 1.41; 3.76) and TH BMD (difference between groups of 2.13%, 95% CI 1.34; 2.92) in the ibandronate group compared to the placebo group.

### Effects of other treatments versus placebo on BMD

#### Denosumab

Two RCTs, including 242 men with osteoporosis [38] and 47 men with osteoporosis respectively followed participants for two years [31] comparing denosumab to placebo. Both studies provided patients in the intervention arm with subcutaneous injection of 60 mg of Denosumab every 6 months. A significant improvement of all BMD sites was observed in the treated group compared to placebo. A MD of 5.80% (95% CI 3.5;8.1) (I^2^ = 78%, p = 0.03) was found for LS BMD, a MD of 2.28% (95% CI 1.51;3.04) (I^2^ = 16%, p = 0.27) was found for TH BMD and a MD of 2.07% (95% CI 1.23;2.92) (I^2^ = 0%, p = 0.85) for FN BMD (Appendix A4).

#### Teriparatide

Two studies, including 309 men with osteoporosis, compared teriparatide treatment to placebo [37, 45]. In the study of Orwoll et al. [37], patients were randomized to receive either daily subcutaneous injection of 20 µg of teriparatide (n = 151) or placebo (n = 147) (a third group receiving 40 µg of teriparatide (n = 139) was also included in the study but not used in our analyses as it is an uncommon dose of treatment) over two years. However, he study was stopped after 11 months. In the study of Kurland et al. [45], patients were randomized to receive either teriparatide (400 IU of parathyroid hormone acid fragment of human PTH (1–34)) (n = 13) or placebo (n = 10) during 18 months. Using meta-analytic statistics, a significant increase of LS BMD was found with teriparatide (MD 8.19, 95% CI 1.14;15.25) as well as a significant increase of FN BMD (MD 1.33, 95% CI 0.39;2.27) (Appendix A5). TH BMD was only reported in the study of Orwoll et al. and authors reported non-significant effect on TH BMD (+ 1.17% for teriparatide group vs + 0.54% for the placebo group, p = NS).

#### Abaloparatide

Two recent studies, including 248 men with osteoporosis, compared abaloparatide (80 µg daily) to placebo [42, 51] administered either during 1-year [42] or 18 months[51]. A significant improvement of all BMD sites was observed in the treated group compared to placebo. A MD of 11.29% (95% CI 1.80; 20.8) (I^2^ = 77%, p = 0.04) was found for LS BMD, a MD of 3.91% (95% CI 0.34; 7.49) (I^2^ = 95%, p < 0.01) for TH BMD and a MD of 3.98% (95% CI 1.10; 6.85) (I^2^ = 69%, p = 0.07) was found for FN BMD (Appendix A6).

#### Romosozumab

One study, including 245 men with osteoporosis compared romosozumab treatment to placebo during 12 months [29]. A monthly injection of 210 mg of romosozumab was provided to 163 men compared to placebo for 82 men. Results reported a mean percentage change from baseline in the LS BMD, TH and FN BMD significantly greater for the romosozumab group compared to the placebo group (LS, + 12.1% vs + 1.2%; TH, + 2.5% vs − 0.5%; FN, + 2.2% vs − 0.2%; all p < 0.001).

### Head-to-head comparisons and effects on BMD

Four studies provided results of head-to-head comparisons. In two of them, teriparatide was compared to alendronate [36, 48], another compared teriparatide to risedronate [41] and the last compared alendronate to zoledronic acid [35].

In both studies comparing teriparatide (20 µg daily in the study of Qi et al. [36] and 40 µg daily in the study of Finkelstein et al. [48]) to alendronate (10 mg daily), authors reported a higher increase of LS BMD in the teriparatide group compared to alendronate (meta-analysis not feasible due to lack of quantitative data available). Finkelstein et al. [48] also measured impact of treatments on FN BMD and reported a significantly higher improvement of FN BMD for the teriparatide group as well.

In the study of Walker et al. [41], comparing a 18-month treatment with 35 mg weekly of risedronate (n = 10) to 20 µg daily of teriparatide (n = 9) (or a combination of both, n = 10), a significant increase of LS BMD was only noticed after the 18-month intervention. However, no significant difference between groups was mentioned. TH and FN BMD only increase significantly in the combination group after 18 months and this increase was significantly superior than the two other groups (p < 0.05).

In the study of Orwoll et al. [35], comparing a 2-year treatment with 5 mg yearly of zoledronic acid (n = 148) to 70 mg daily of alendronate (n = 158), authors reported an increase of LS BMD, TH BMD and FN BMD in both groups over 24-months. The noninferiority of zoledronic acid versus alendronate was established but superiority was not demonstrated.

### Effects of treatments on fractures

Incidence of fractures (i.e. either vertebral or non-vertebral fractures) was reported in 16 studies (76%), but only four studies defined incidence of fracture as the primary outcome [30, 31, 39, 44]. In the study of Boonen et al. 2012 [44] including 1199 men with osteoporosis, authors observed that zoledronic acid provided during 12 months significantly reduced the incidence of fractures observed during 24-months of follow-up compared to placebo. The rate of any new morphometric vertebral fracture was 1.6% in the zoledronic acid group and 4.9% in the placebo group over the 24-month period, representing a 67% risk reduction with zoledronic acid (relative risk, 0.33; 95% confidence interval, 0.16 to 0.70; P = 0.002). In another study of Boonen et al. 2011 [30], a sample of 508 men with recent hip fracture was also randomized to either zoledronic acid or placebo. No significant difference between groups was found in regards of the rate of clinical fracture at month 24 (i.e. 7.5% in the zoledronic acid group vs 8.7% in the placebo group, p = 0.64). In the third study including 47 men with osteoporosis, the one of Nakamura et al. [31], authors reported no new vertebral fracture in men treated with denosumab versus an incidence of 8.3% in the placebo group. The difference between groups was not significant (p = 0.15). The fourth study, published by Ringe et al. 2009 [39] included 158 men with risedronate treatment during 2 years and 158 men with placebo. Authors reported a significantly higher incidence of fractures in the placebo group compared to the risedronate group (i.e. incidence of VF fractures of 23.6% versus 9.2%, p = 0.003; incidence of NVF of 22.3% versus 11.8%, p = 0.03). All other studies were not powered for this outcome. In most of included studies, authors simply reported the number of fractures in each group, without any statistical comparisons between them. Incidence of fractures was generally very low (median of VF 1.7% in the treatment group versus 4.1% in the PBO group across studies; median of NVF 3.8% in both groups across studies). None of the 16 included studies reported a higher risk of fractures in the treatment group compared to placebo. No safety issue in regards of the risk of fracture was therefore reported for any of the treatments.

Results of incidence of fracture within each of the 16 studies are reported in Table 3.

**Table 3:** Summary of the incidence of fractures

First author	Participants (n) and Treatment/comparator	Study duration	Primary/secondary endpoint	Type of fractures reported	Number of fractures treatment group	Number of fracture control group
Boonen 2009 [43]	Risedronate (n = 191)/Placebo (n = 93)	24 months	Secondary endpoint	VF + all clinical fractures	All clinical fractures: 7 VF: 2	NVF: 6 VF: 0
Boonen 2011 [30]	Zoledronic acid (n = 248)/Placebo (n = 260)	24 months	Primary endpoint	All fractures	Incident fractures: 16	Incident fractures: 20
Boonen, 2012 [44]	Zoledronic acid (n = 588)/Placebo (n = 611)	24 months	Primary endpoint	VF	VF: 9	VF: 28
Czerwinski 2022 [42]	Abaloparatide (n = 174)/Placebo (n = 64)	12 months	Secondary endpoint	VF + NVF	NVF: 1 VF: 0	NVF: 2 VF: 1
Kurland 2000 [45]	Teriparatide (n = 13)/Control (n = 10)	18 months*	Secondary endpoint	VF	VF: 1	VF: 2
Lewiecki 2018 [29]	Romosozumab (n = 163)/Placebo (n = 82)	12 months	Safety	All fractures	Incident fractures: 3	Incident fractures: 2
Miller 2004 [32]	Alendronate (n = 109)/Placebo (n = 58)	12 months	Safety	VF + NVF	NVF: 6 Morphometric VF: 6 Clinical VF: 5	NVF: 1 Morphometric VF: 3 Clinical VF: 3
Nakamura 2014 [31]	Denosumab (n = 23)/Placebo (n = 24)	24 months	Primary endpoint	VF	VF: 0	VF: 2
Orwoll 2000 [34]	Alendronate (n = 146)/ Placebo (n = 95)	24 months	Secondary endpoint	VF + NVF	NVF: 6 VF: 1	NVF: 5 VF: 7
Orwoll, 2003 [37]	Teriparatide (n = 151)/Placebo (n = 147)	12 months	Safety	NVF	NVF: 2	NVF: 3
Orwoll, 2010 [35]	Zoledronic acid (n = 154)/Alendronate (n = 148)	24 months	Secondary endpoint	VF	VF: 4	VF: 6
Orwoll, 2010 [33]	Ibandronate (n = 85)/Placebo (n = 47)	12 months	Safety	VF + Clinical fractures	VF: 1 Clinical fracture: 3	VF: 2 Clinical fracture: 0
Orwoll, 2012[38]	Denosumab (n = 111)/Placebo (n = 117)	12 months	Secondary endpoint	VF + NVF	NVF: 1 VF: 0	NVF: 1 VF: 1
Ringe, 2009 [39]	Risedronate (n = 158)/Control (n = 158)	24 months	Primary endpoint	VF + NVF	NVF: 18 VF: 14	NVF: 33 VF: 35
Shimon, 2005 [40]	Alendronate (n = 11)/Placebo (n = 13)	12 months	Safety	VF + NVF	NVF:0 VF:0	NVF:1 VF:1
Walker, 2013[41]	Risedronate (n = 10)/Teriparatide (n = 9)/Combined (n = 10)	18 months	VF: Secondary endpoint Clinical F: safety	VF + Clinical fractures	VF: 1 Clinical fracture (12 months): 0	Teriparatide: VF: 0; Clinical fracture (12 months): 0 Combined: VF:1; Clinical fractures (12 months): 1

* Fractures assessed at 12 months

*VF* vertebral fracture, *NVF* non vertebral fractures, *F* fractures

### GRADE assessment

GRADE assessments is available in Appendix A7. GRADE level of evidence was attributed for all the 6 meta-analyses run (i.e. effects of any bisphosphonate versus placebo, alendronate versus placebo, risedronate versus placebo, denosumab vs placebo, teriparatide versus placebo and abaloparatide versus placebo on BMD). Risk of bias was considered as not serious in all studies included in those meta-analyses; publication bias was only measured in one meta-analysis run (i.e. any bisphosphonates versus placebo) because all other meta-analyses included a too restricted number of studies; inconsistency (i.e. unexplained heterogeneity) was observed for most of the meta-analyses; serious imprecision was considered for all meta-analysis comprising only 2 or 3 studies and no serious indirectness was considered for all meta-analyses. A high level of evidence was considered for the efficacy of any bisphosphonate on BMD (), a moderate level of evidence was considered for the efficacy of denosumab, alendronate and risedronate on BMD () and, finaly, a low level of evidence was considered for the efficacy of teriparatide and abaloparatide on BMD ().

## Discussion

This systematic review and meta-analysis provides evidence that alendronate, risedronate, zoledronic acid, ibandronate, denosumab, teriparatide abaloparatide and romosozumab all have a beneficial effect on lumbar spine, total hip and femoral neck BMD of men suffering from osteoporosis, as compared with placebo. The strength of evidence is however limited by the low number of studies included in the analyses and the unexplained heterogeneity observed in some comparisons. Fracture data in men are scant at all sites (vertebral, and non-vertebral fractures) and few randomized controlled studies have reported the efficacy of pharmacological treatment on the incidence of fracture as the primary endpoint. Hence, the efficacy of these treatments to reduce the incidence of fracture is still inconclusive. Nevertheless, as previously mentioned, regulatory agency guidance indicate that, once an indication in postmenopausal women as been granted, a separate bridging study based on changes in BMD can be sufficient to grant an authorization for men with osteoporosis [16, 17]. Bone mineral density is suggested as a “surrogate marker” in studies of men [18, 19], it is therefore not surprising that our systematic literature search identified a larger number of studies using BMD rather than fracture incidence as the primary outcome.

A previous systematic review and meta-analysis [21] also reported efficacy of osteoporosis treatment on BMD of men with osteoporosis. Authors included 13 randomized controlled studies published until 2014. Despite using slightly different inclusion criteria from ours and including a lower number of trials, they also confirmed the efficacy of most of the proposed treatment in improving BMD at the lumbar spine. Other BMD sites were not investigated. In the previous study, the authors also provided a hierarchy of treatments using network meta-analytic statistics. This methodology can however be discussed as the authors only included one study for the majority of comparisons investigated. This is the main reason why we decided not to perform a network meta-analysis on our data. Nevertheless, when comparing the effect sizes found in all of our analyses, abaloparatide seems to be the most effective treatment proposed for the increase of lumbar spine BMD. The effect size for abaloparatide (i.e. MD of 11.29) is superior to the effect size of teriparatide (i.e. MD of 8.19), which is in line with previous published data [12]. These assumptions are, however, only observational as providing a hierarchy between treatments was not the objective of the current study. Another systematic review and meta-analysis, published by Nayak et al. [22] reported efficacy of treatments on vertebral and non-vertebral fractures directly. Authors included 22 individual studies and revealed that alendronate and risedronate are effective in reducing vertebral fractures. However, they did not report significant effect of denosumab. One important point to highlight is, once again, that a very limited number of studies were included in each forest plot (e.g. only two studies for alendronate versus placebo and two studies for risedronate versus placebo comparisons). Pooling all bisphosphonates (4 studies respectively for vertebral and non-vertebral fractures risk association), the authors confirmed the beneficial effects of this treatment category to reduce the risk of both vertebral and non-vertebral fractures. This being said, our results are in line with those two previous meta-research studies as both of them demonstrated the efficacy of treatments either on BMD or on fracture risk.

Overall, our results also support current guidelines [1, 52, 53] to use bisphosphonates (alendronate, risedronate, ibandronate and zoledronate) [54] and denosumab in patients with high risk of fracture and teriparatide in patients with very high risk of fractures. Nevertheless, these guidelines have been developed for postmenopausal women with osteoporosis and specific guidelines for the management of men with high risk/very high risk of fractures may be developed by, among other strategies, taking the results of this present meta-analysis into consideration.

There are some strengths in this paper. Most importantly, we followed the best practices to conduct a systematic literature review and meta-analysis. As example, we used PRISMA2020 checklist [23] for the completeness of reporting, AMSTAR2 quality appraisal tool [55] to ensure a high-quality level to our methodology, Cochrane Handbook for systematic literature review and meta-analysis to guarantee a high-quality level to our meta-analytical statistics [27]. Also, we and used a comprehensive search strategy to minimize the possibility of publication bias. As a result of this, no publication bias was found in this systematic review and meta-analysis. This strategy was preferred to highlight the efficacy of treatment on osteoporosis alone. Our work nevertheless contains several limitations. First, a limited number of studies were included in the different forest plots, which prevented subgroup analyses exploring potential sources of heterogeneity, sensitivity analyses or publication bias analyses. This is particularly true for denosumab, abaloparatide and teriparatide for which only two randomized controlled trials met our inclusion criteria and were combined in a meta-analytic statistical model. For romosozumab, only one study was identified and no meta-analysis was run. Given the restricted number of studies available to measure the efficacy of these different treatments for osteoporosis in men, the strength of evidence is considered to be moderate to low. The second limitation of this work concerns the length of treatment that may vary from one study to another. In the forest plots, we combined studies that used various length of treatment, which is not optimal. Moreover, in the study of Hwang et al. patients were treated with alendronate during only 6 months. Even if participants improved their LS and FN BMD during this period, the non-significant effect on TH BMD could be partially explained by this short period of treatment. Third, it is regrettable that a very restricted number of studies used the incidence of fracture as primary outcome. In studies measuring the efficacy of pharmacological treatment on fracture incidence but defining fracture as a secondary outcome or a safety marker, the sample size and length of study may not be appropriate to detect a significant difference, if this exists. Therefore, it was considered inappropriate to run a meta-analysis on this outcome and the results were only presented narratively. Fourth, the majority of the clinical trials (i.e. 14 out of 21, 66.7%) included in this meta-analysis used the increase of lumbar spine bone mineral density as the primary outcome measure. Currently, studies aiming to validate change in bone mineral density as a surrogate endpoint for fracture outcomes mainly focused on hip bone mineral density. Stronger associations have been found between change in total hip BMD and incidence of vertebral and hip fractures [18, 19]. Nevertheless, these observations do not mean that using lumbar spine BMD increase as surrogate marker for fracture risk is irrelevant, as Bouxsein et al. [56] reported similar correlations values for vertebral fracture and change in lumbar spine, total hip and femoral neck BMD in a meta-regression including 38 placebo-controlled trials.

As previously mentioned, the European regulatory authorities accept the use of bone mineral density (BMD) as a primary outcome in pivotal studies evaluating the efficacy of chemical entities intended for the management of osteoporosis in males, providing these chemical entities were approved for the management of osteoporosis in females, based on a pivotal study showing a reduction in fracture rates. Total hip or femoral neck are widely recognized as the most appropriate measurement sites for BMD, in such studies (bridging studies). Nevertheless, a minimal duration of 12 months for bone forming agents and of 24 months for anti-resorptive agents is usually considered as a minimal length of treatment. In the future, the assessment of biochemical markers of bone remodeling could be further investigated to see whether CTX1 for anti-resorptive agents and PINP for bone-forming agents could also be considered as surrogate markers for anti-fracture efficacy, in the context of bridging studies.

## Conclusion

Through a systematic review and meta-analysis including 21 randomized controlled trials, we have established that medications used in the management of osteoporosis in women (i.e. alendronate, risedronate, zoledronic acid, ibandronate, denosumab, teriparatide abaloparatide and romosozumab) appear to be similarly beneficial in men with osteoporosis. Therefore, the algorithm for the management of osteoporosis in men could be identical to that recommended for the management of osteoporosis in women.

## Supplementary Information

Below is the link to the electronic supplementary material.

Supplementary file1 (DOCX 264 KB)

## Data Availability

The databases and R script for running meta-analyses have been deposit on Open Science Framework (https://osf.io/wqy3n/).
