# Supplementary material for: Efficacy of osteoporosis pharmacological treatments in men: a systematic review and meta-analysis
Source: Aging Clin Exp Res. 2023 Jul 3;35(9):1789–806. doi: 10.1007/s40520-023-02478-9 (PMC10460304; doi:10.1007/s40520-023-02478-9)
Supplement: Supplementary file 1 — Supplementary file1 (DOCX 264 KB) [file 40520_2023_2478_MOESM1_ESM.docx]

**Appendices**

**Efficacy of osteoporosis treatments in men: a systematic review and meta-analysis**

**Authors**

Charlotte Beaudart^1^, Céline Demonceau^1^, Shaun Sabico^2^, Nicolas Veronese^3^, Cyrus Cooper^4^, Nicholas Harvey^4^, Nicholas Fuggle^4^, Olivier Bruyère, René Rizzoli^5^, Jean-Yves Reginster^1^

**Affiliations**

1 WHO Collaborating Center for Public Health aspects of musculo-skeletal health and ageing, Division of Public Health, Epidemiology and Health Economics, University of Liège, Belgium

2 Biochemistry Department, College of Science, King Saud University, Riyadh P.O. Box 11451, Saudi Arabia.

3 Geriatric Unit, Department of Internal Medicine, Geriatrics Section, University of Palermo, via del Vespro, 141, 90127, Palermo, Italy

4 MRC Lifecourse Epidemiology Centre, University of Southampton, Southampton, SO16 6YD, UK

5 Service of Bone Diseases, Geneva University Hospitals and Faculty of Medicine, Geneva, Switzerland

Appendix A1. Search strategies

| **Database: Ovid MEDLINE(R) ALL <1946 to May 22, 2023>  Search Strategy:**  1  Osteoporosis/ (50413)  2  Bone Diseases, Metabolic/ (9018)  3  Bone Density/ (60551)  4  Bone Demineralization, Pathologic/ (372)  5  Osteoporotic Fractures/ (7927)  6  osteoporo*.ti,ab,kf. (92900)  7  osteopenia*.ti,ab,kf. (11052)  8  (bone* adj1 (densit* or content)).ti,ab,kf. (22043)  9  (bone* adj1 (demineralization or demineralisation of loss* or decreas* or deterioration*)).ti,ab,kf. (6934)  10  metabolic bone disease*.ti,ab,kf. (3246)  11  (osteoporo* adj1 fracture*).ti,ab,kf. (9275)  12  or/1-11 (152461)  13  (denosumab or alendronate or risedronate or abaloparatide or teriparatide or ibandronate or zoludronate or biphosphonate* or romosozumab or zoledronic).ti,ab,kf. (16263)  14  alendronate/ or ibandronic acid/ or risedronic acid/ or zoledronic acid/ (9197)  15  13 or 14 (18326)  16  (men or man or male* or boy* or masculin).ti,ab,kf. (2345661)  17  12 and 15 and 16 (1266)  18  Case Reports/ (2336495)  19  "Systematic Review"/ (228942)  20  exp "review"/ (3161084)  21  Meta-Analysis/ (181190)  22  protocol*.ti. (91582)  23  or/18-22 (5602875)  24  17 not 23 (801) |
| --- |
| **Database: EBM Reviews - Cochrane Central Register of Controlled Trials <April 2023>  Search** **Strategy:**  1  Osteoporosis/ (3599)  2  Bone Diseases, Metabolic/ (635)  3  Bone Density/ (6195)  4  Bone Demineralization, Pathologic/ (16)  5  Osteoporotic Fractures/ (498)  6  osteoporo*.ti,ab,kf. (10318)  7  osteopenia*.ti,ab,kf. (1200)  8  (bone* adj1 (densit* or content)).ti,ab,kf. (3411)  9  (bone* adj1 (demineralization or demineralisation of loss* or decreas* or deterioration*)).ti,ab,kf. (693)  10  metabolic bone disease*.ti,ab,kf. (178)  11  (osteoporo* adj1 fracture*).ti,ab,kf. (1080)  12  or/1-11 (16634)  13  (denosumab or alendronate or risedronate or abaloparatide or teriparatide or ibandronate or zoludronate or biphosphonate* or romosozumab or zoledronic).ti,ab,kf. (5256)  14  alendronate/ or ibandronic acid/ or risedronic acid/ or zoledronic acid/ (2013)  15  13 or 14 (5448)  16  (men or man or male* or boy* or masculin).ti,ab,kf. (210692)  17  12 and 15 and 16 (453) |


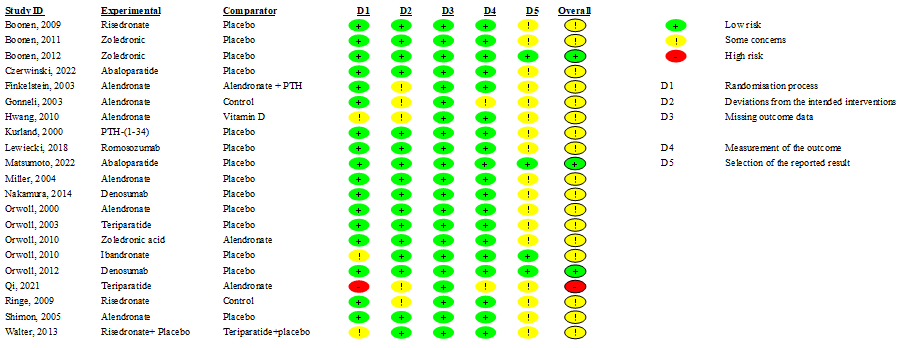


**Appendix A2**. Risk of Bias Assessment (Cochrane ROB2 Tool) for the 21 included RCTs


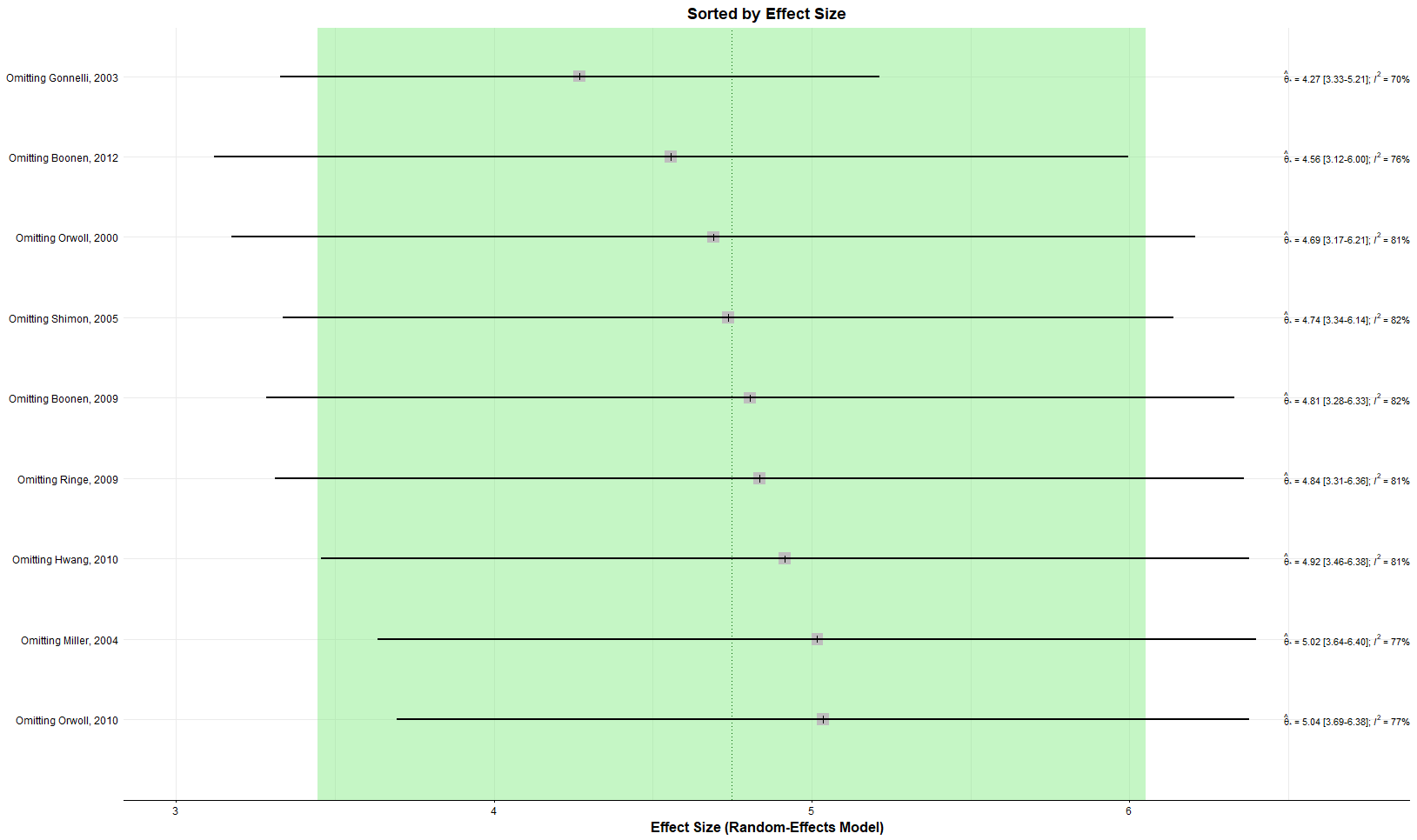


(A) One-Leave-Out analysis – Bisphosphonates and Lumbar Spine BMD


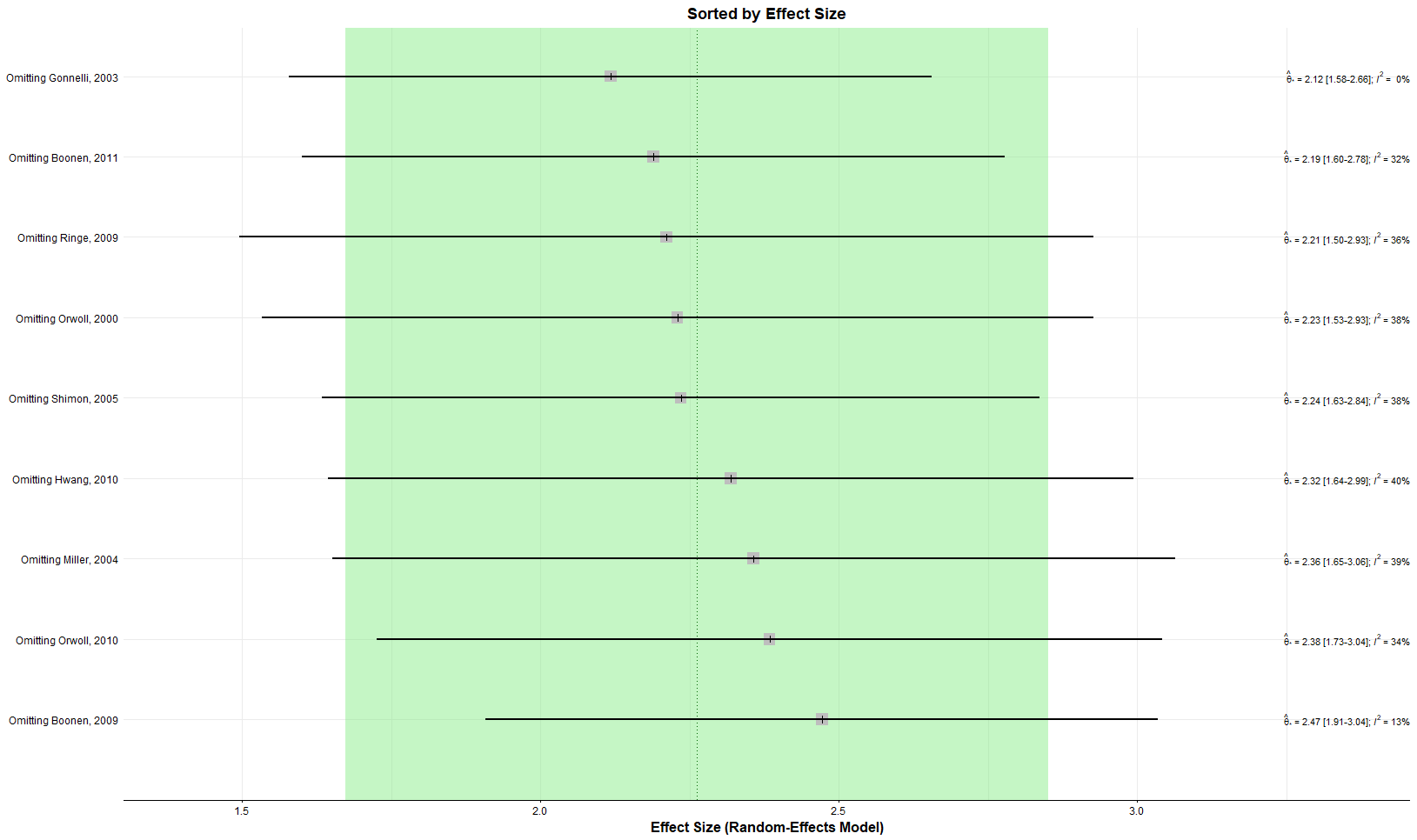


(B) One-Leave-Out analysis – Bisphosphonates and Femoral Neck BMD


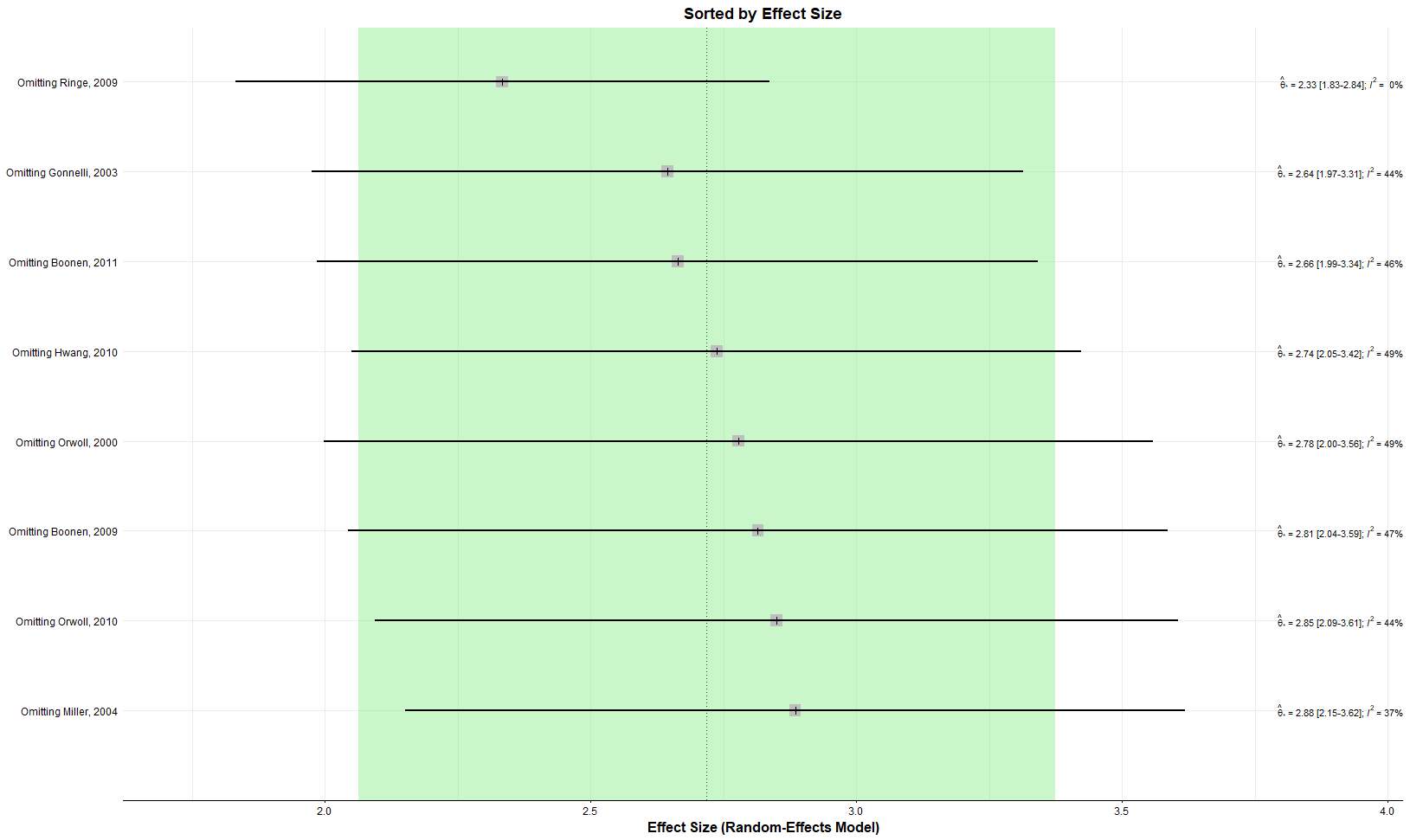


(C) One-Leave-Out analysis – Bisphosphonates and Hip BMD

**Appendix A3**. One-leave-Out analyses – Bisphosphonates treatment for men with osteoporosis


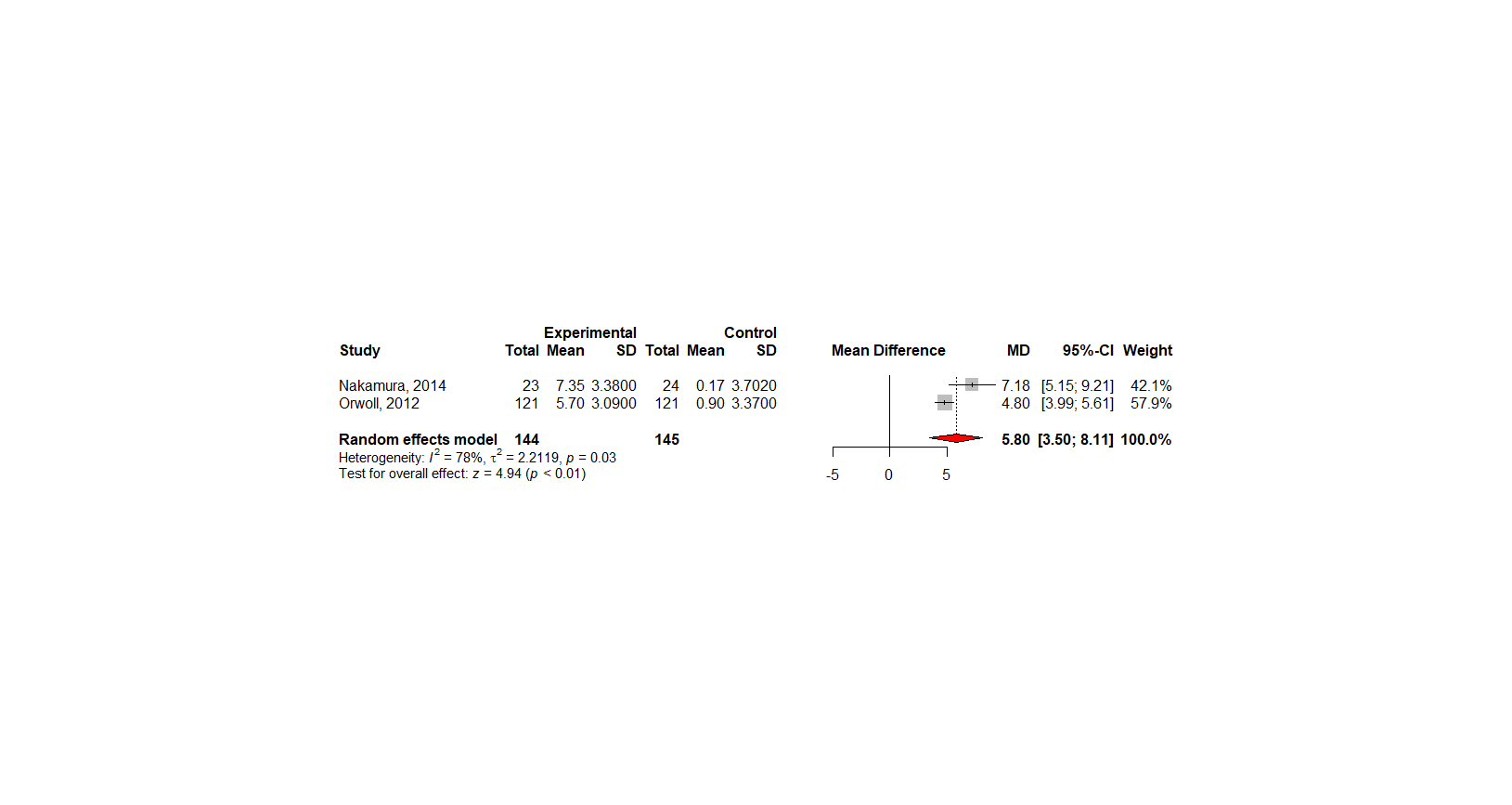


(A)


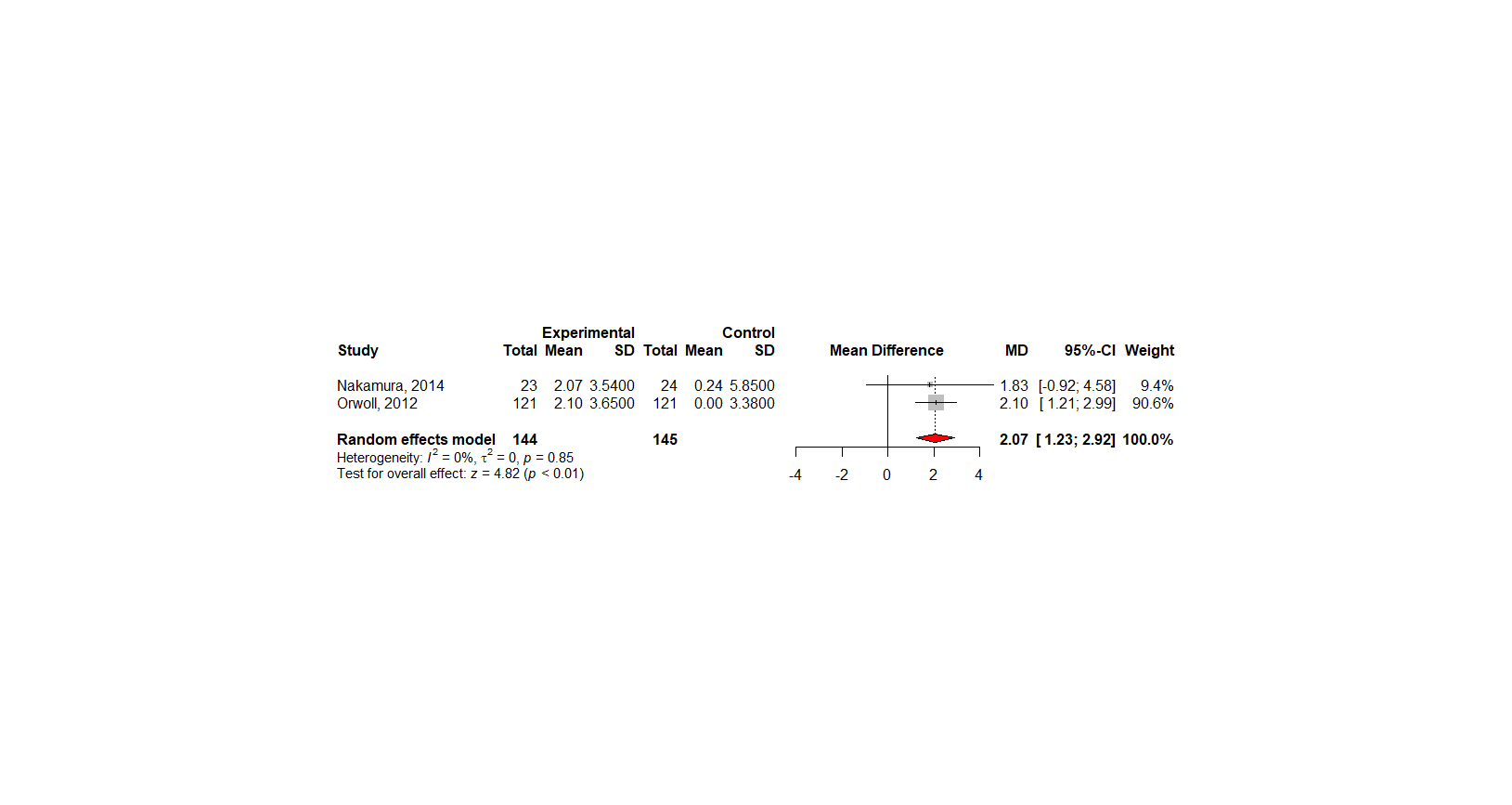


(B)


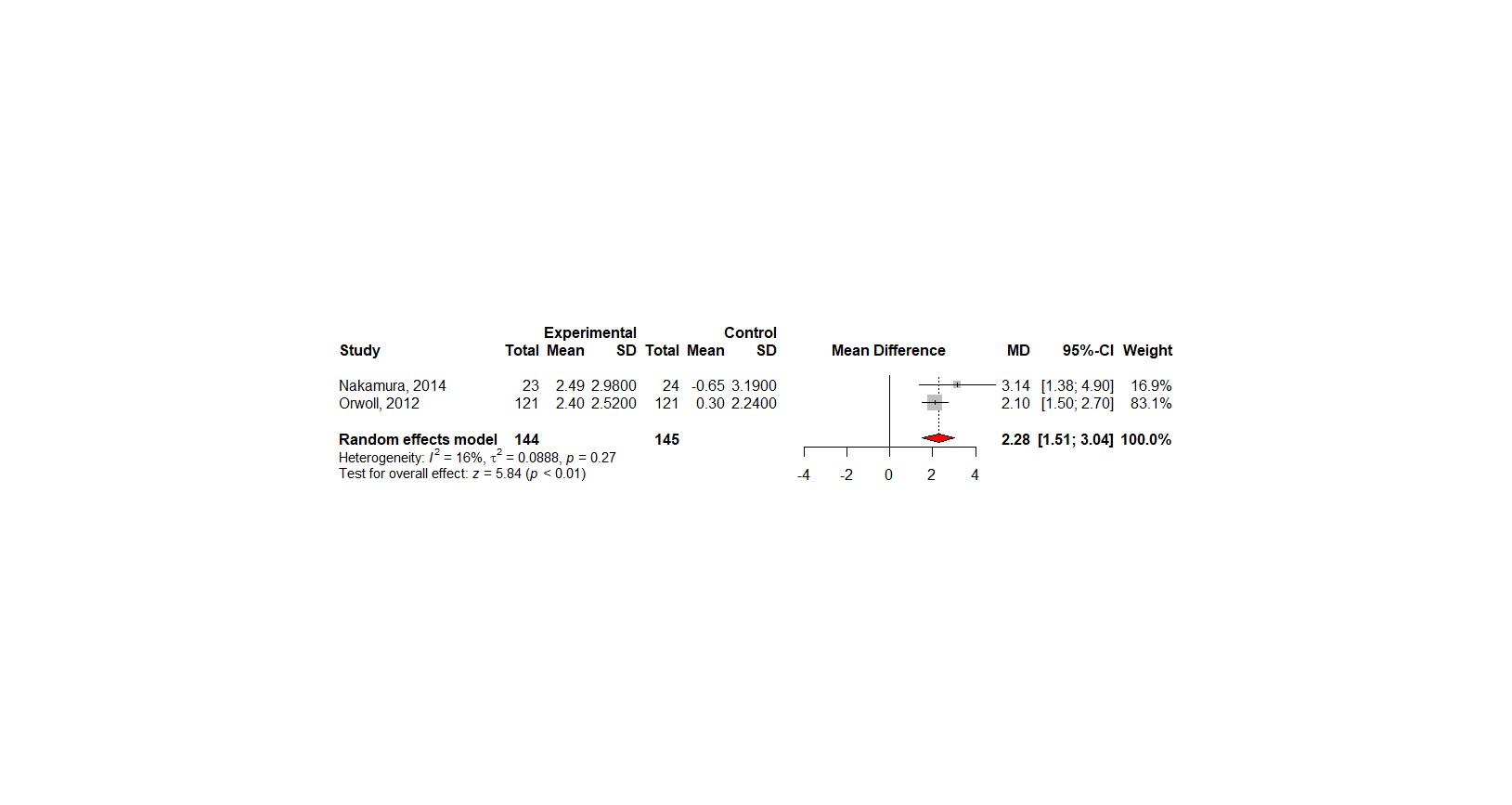


(C)

**Appendix A4.** Effects of Denosumab vs placebo on (A) LS BMD and (B) FN BMD and (C) TH BMD


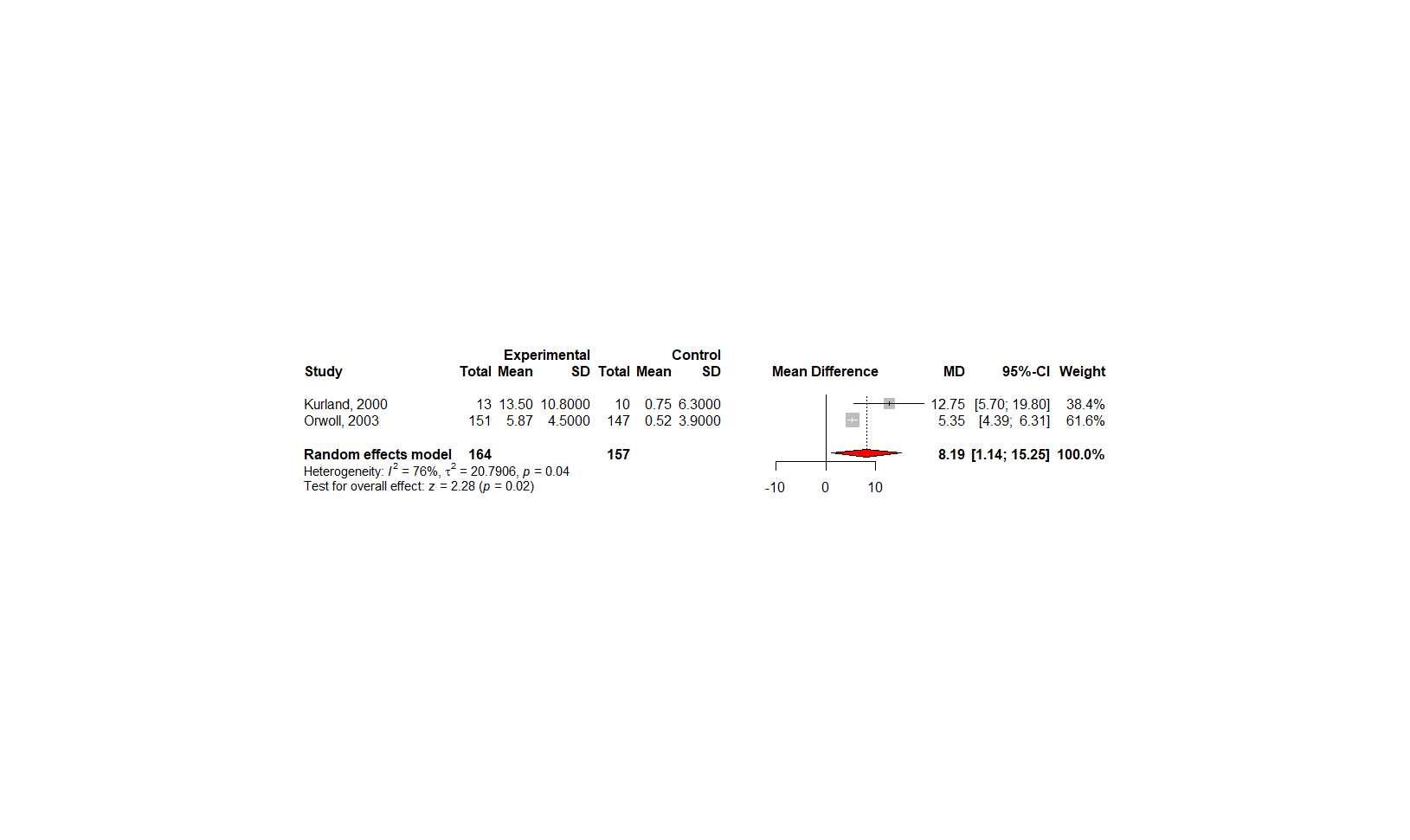


(A)


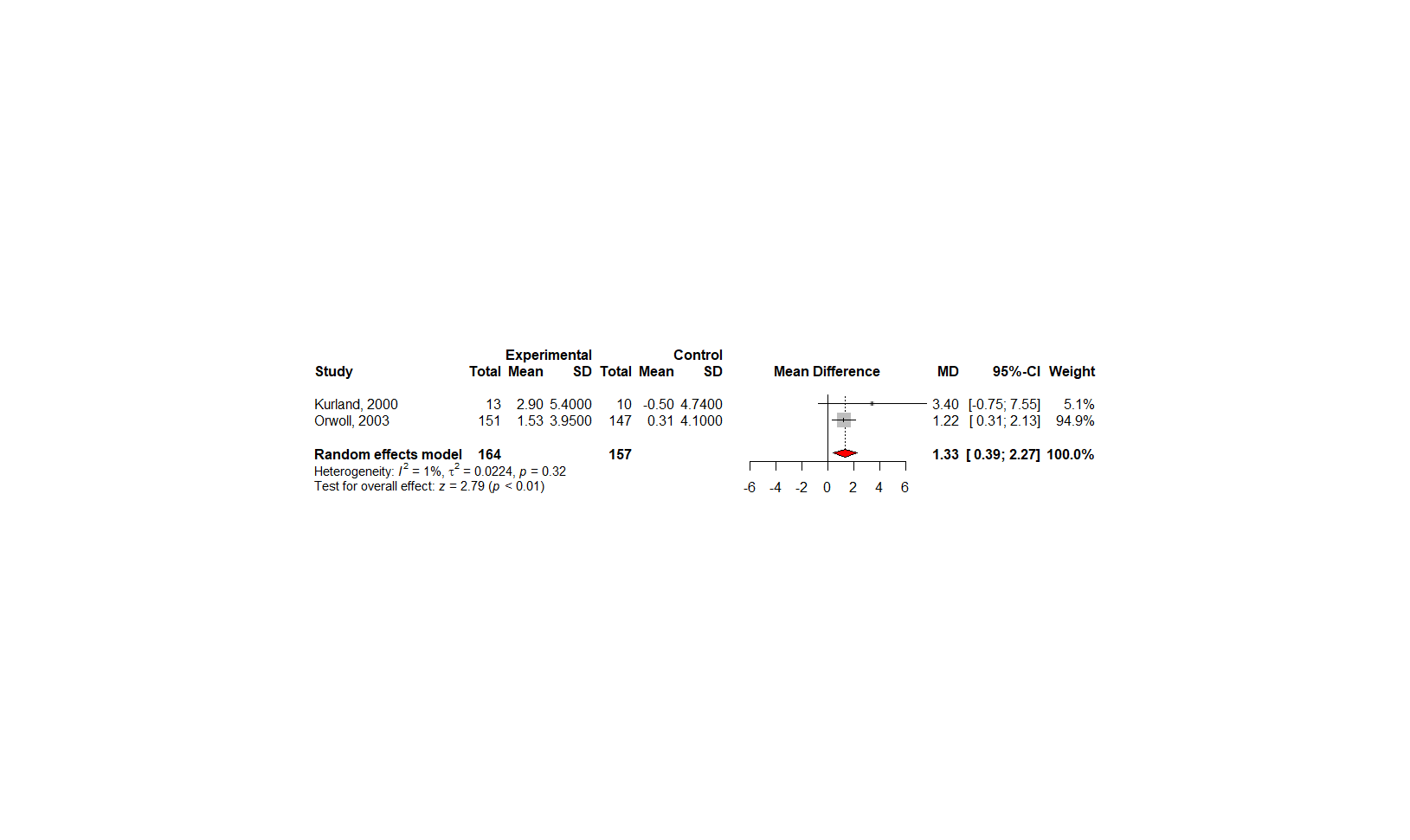


(B)

**Appendix A5.** Effect of Teriparatide vs placebo on (A) LS BMD and (B) FN BMD


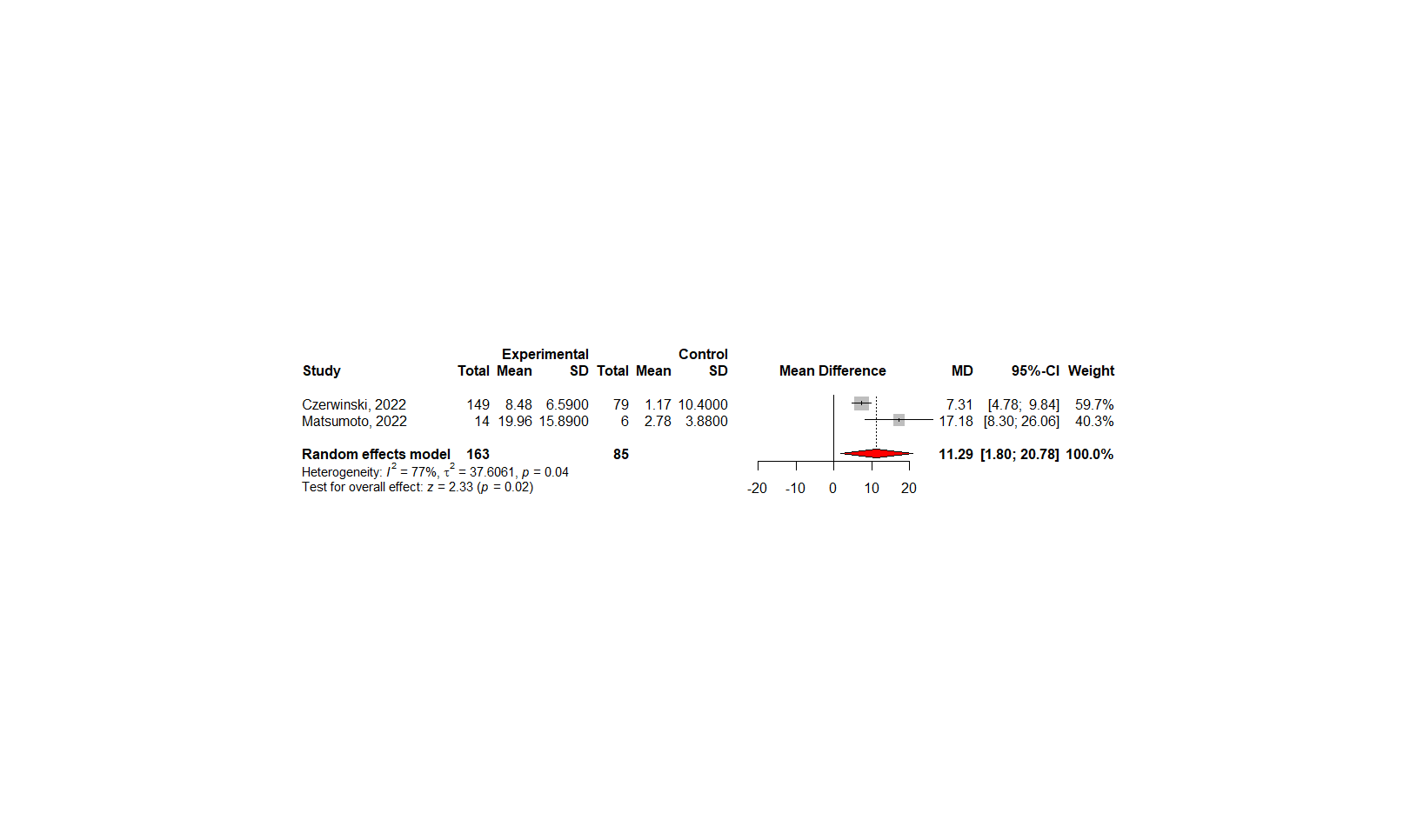


(A)


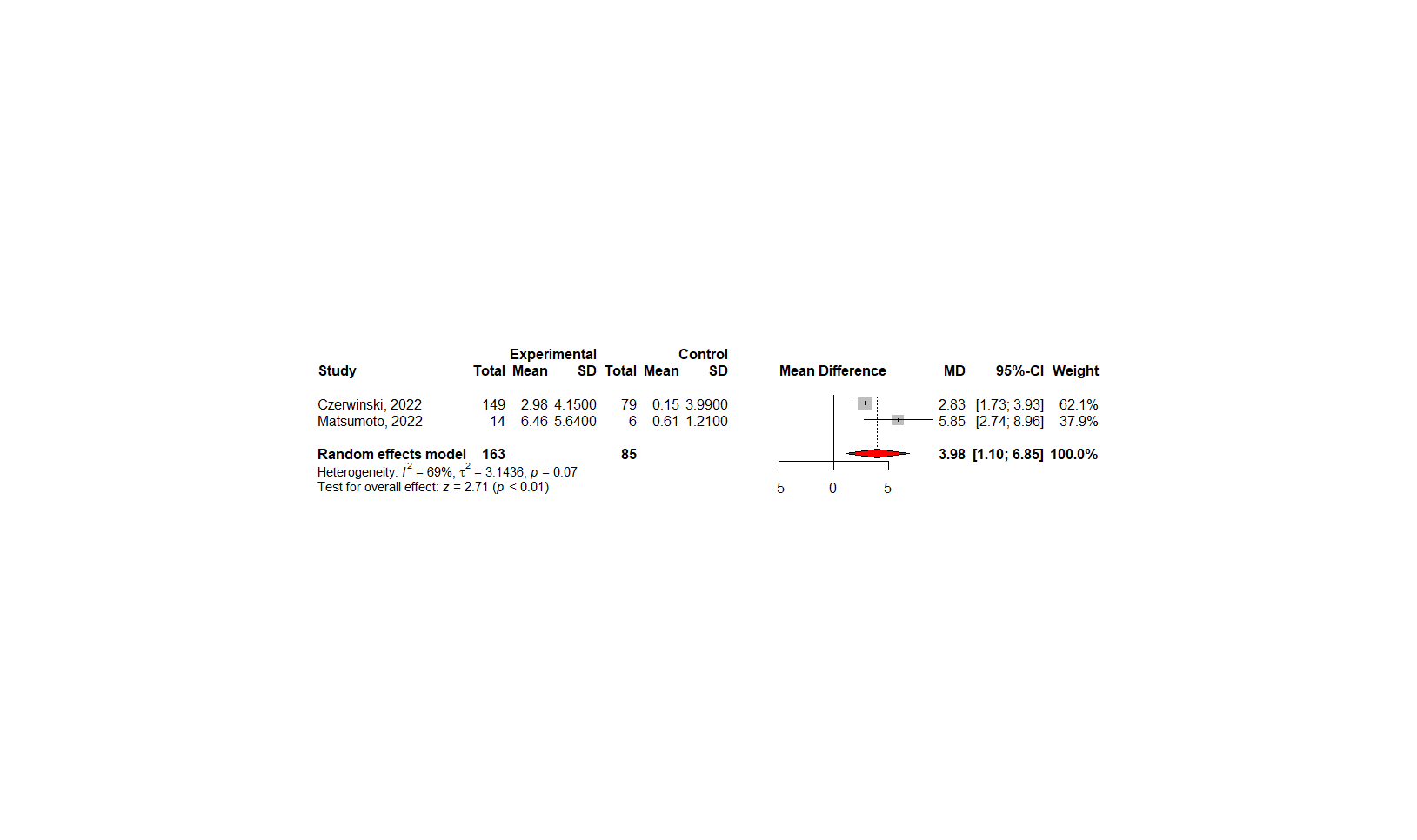


(B)


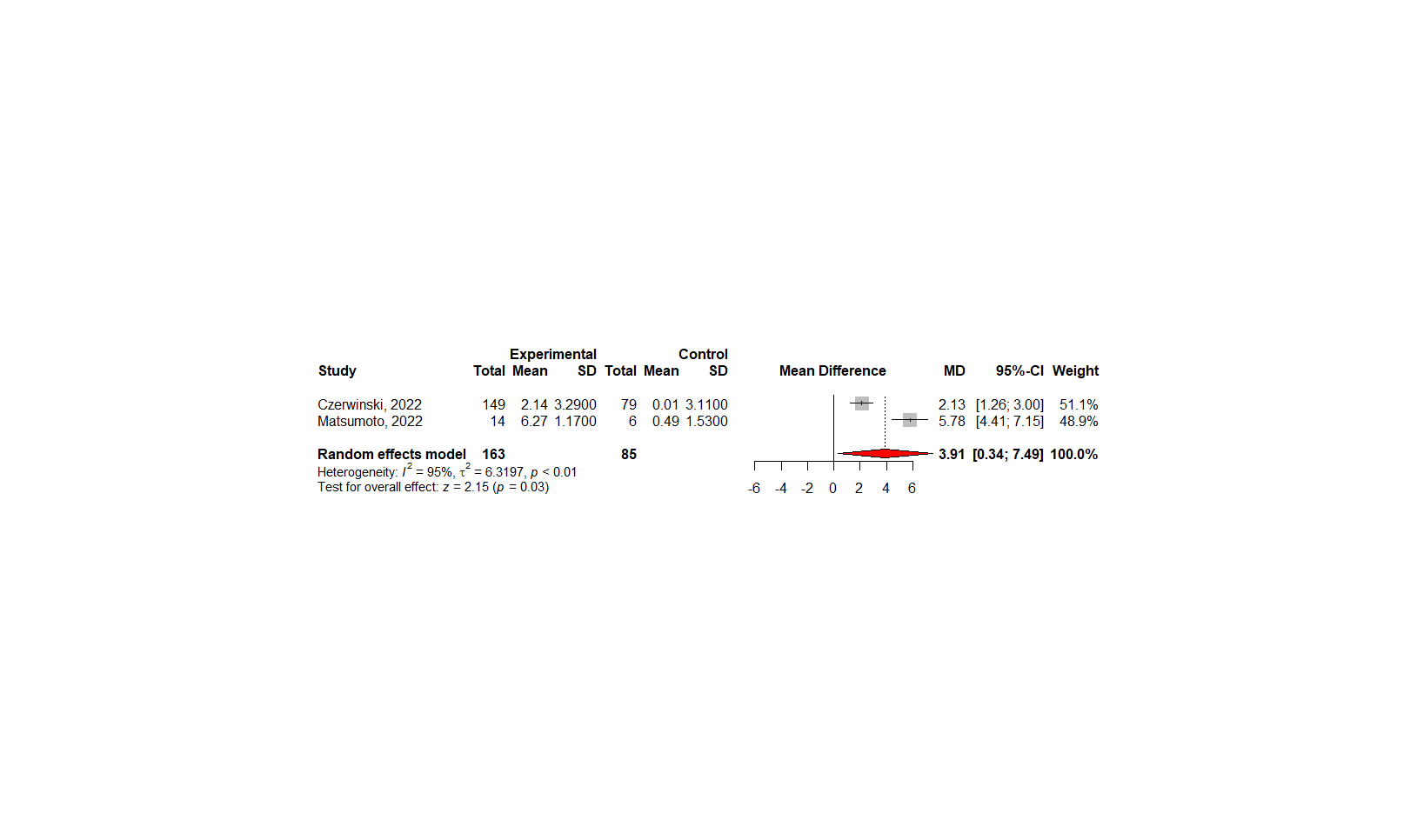


(C)

**Appendix A6.** Effect of Abaloparatide vs placebo on (A) LS BMD and (B) FN BMD and (C) TH BMD

**Appendix A7.** Summary of qualitative or quantitative reporting of results

| Treatments | n. studies / no of participants | Outcomes | Quantitative analysis possible | | | GRADE assessment | | | | | |
| --- | --- | --- | --- | --- | --- | --- | --- | --- | --- | --- | --- |
|  |  |  | MA | Leave-one-out sensitivity analysis | Publication bias assessment | Risk of bias | Inconsistency | Indirectness | Imprecision | Publication bias | Overall certainty of evidence |
| ***Bisphosphonates versus placebo*** | | | | | | | | | | | |
| Alendronate vs placebo | 5 studies  553 patients | *BMD:* Lumbar spine BMD (n=5), total Hip BMD (n=4), femoral neck BMD (n=5) | *YES* | *YES* | *NO* | *Not serious* | *Serious* | *Not serious* | *Not serious* | *Not measurable* | *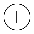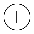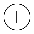* |
|  |  | *Fractures:* Vertebral fractures (n=3), non vertebral fractures (n=3) | *NO* | *NO* | *NO* | *NA* | *NA* | *NA* | *NA* | *NA* | *NA* |
| Risedronate vs placebo | 2 studies  600 patients | *BMD:* Lumbar spine BMD (n=2), femoral neck BMD (n=2), hip BMD (n=2) | *YES* | *NO* | *NO* | *Not serious* | *Not serious* | *Not serious* | *Serious* | *Not measurable* | *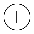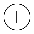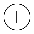* |
|  |  | *Fractures:* Vertebral fractures (n=2), non vertebral fractures (n=2) | *NO* | *NO* | *NO* | *NA* | *NA* | *NA* | *NA* | *NA* | *NA* |
| Ibandronate vs placebo | 1 study  132 patients | *BMD:* Lumbar spine BMD (n=1), Femoral Neck BMD (n=1), Hip BMD (n=1)  *Fractures :* vertebral fractures (n=1) | *NO* | *NO* | *NO* | *NA* | *NA* | *NA* | *NA* | *NA* | *NA* |
| Zoledronic acid vs placebo | 2 studies  1,707 patients | *BMD:* Lumbar Spine (n=1), Femoral neck BMD (n=1), total hip BMD (n=1)  *Fractures:* vertebral fractures (n=1) | *NO* | *NO* | *NO* | *NA* | *NA* | *NA* | *NA* | *NA* | *NA* |
| Any bisphosphonate vs placebo | 9 studies  2,992 patients | *BMD:* Lumbar spine BMD (n=9), Femoral neck BMD (n=9), total hip BMD (n=8) | *YES* | *YES* | *YES* | *Not serious* | *Serious* | *Not serious* | *Not serious* | *None* | *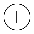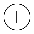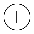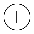* |
|  |  | *Fractures*: vertebral fractures (n=6), non-vertebral fractures (n=6) | *NO* | *NO* | *NO* | *NA* | *NA* | *NA* | *NA* | *NA* | *NA* |
| ***Other treatments versus placebo*** | | | | | | | | | | | |
| Denosumab vs placebo | 2 studies  288 patients | *BMD:* Lumbar spine BMD (n=1), Femoral neck BMD (n=1), total hip BMD (n=1) | *YES* | *NO* | *NO* | *Not serious* | *Not serious* | *Not serious* | *Serious* | *Not measurable* | *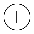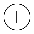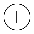* |
|  |  | *Fractures:* vertebral fractures (n=2), non-vertebral fractures (n=2) | *NO* | *NO* | *NO* | *NA* | *NA* | *NA* | *NA* | *NA* | *NA* |
| Teriparatide vs placebo | 2 studies  309 patients | *BMD:* Lumbar spine BMD (n=2), total Hip (n=2) BMD, femoral neck BMD (n=2) | *YES* | *NO* | *NO* | *Not serious* | *Serious* | *Not serious* | *Serious* | *Not measurable* | *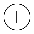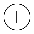* |
|  |  | *Fractures: VF (n=1), NVF (n=1)* | *NO* | *NO* | *NO* | *NA* | *NA* | *NA* | *NA* | *NA* | *NA* |
| Abaloparatide vs placebo | 2 study  248 patients | *BMD:* Lumbar spine BMD (n=2), total Hip BMD (n=2), femoral neck BMD (n=2) | *YES* | *NO* | *NO* | *Not serious* | *Serious* | *Not serious* | *Serious* | *Not measurable* | *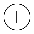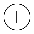* |
|  |  | *Fractures (n=1)* | *NO* | *NO* | *NO* | *NA* | *NA* | *NA* | *NA* | *NA* | *NA* |
| Romosozumab vs placebo | 1 study  245 patients | *BMD:* Lumbar spine BMD, total Hip BMD | *NO* | *NO* | *NO* | *NA* | *NA* | *NA* | *NA* | *NA* | *NA* |
| ***Head-to-head comparisons*** | | | | | | | | | | | |
| Teriparatide vs Alendronate | 2 studies  183 patients | *BMD:* lumbar spine BMD (n=2), total Hip BMD (n=1), femoral neck BMD (n=1) | *NO* | *NO* | *NO* | *NA* | *NA* | *NA* | *NA* | *NA* | *NA* |
| Teriparatide vs Risedronate | 1 study  29 patients | *BMD*: Lumbar spine BMD, total Hip BMD, femoral neck BMD  *Fractures:* vertebral fractures | *NO* | *NO* | *NO* | *NA* | *NA* | *NA* | *NA* | *NA* | *NA* |
| Alendronate vs Zoledronic Acid | 1 study  306 patients | *BMD:* Lumbar spine BMD, total Hip BMD, femoral neck BMD  *Fractures: vertebral fractures* | *NO* | *NO* | *NO* | *NA* | *NA* | *NA* | *NA* | *NA* | *NA* |

NA: not applicable
